# Comparison of two novel options for resuspension of inverted U-shaped nasopharyngeal flap following endonasal access to cranio-vertebral junction: Pilot cadaveric analysis and illustrative case report

**DOI:** 10.1007/s10143-025-04068-x

**Published:** 2026-01-08

**Authors:** Beatrice Zucca, Marissa Koscielski, John Na, Andrea de Gregorio, Ian Smith, Joel Kaye, Ahmad R. Sedaghat, Samer Hoz, Charles J. Prestigiacomo, Rani M. Nasser, Justin N. Virojanapa, Norberto O. Andaluz, Katie M. Phillips, Jonathan A. Forbes

**Affiliations:** 1 Alma Mater Studiorum-University of Bologna, Bologna, Italy; 2 University of Cincinnati College of Medicine, Cincinnati, OH USA; 3 Università di Milano, Milano, Italy; 4 Department of Neurosurgery, University of Cincinnati Medical Center, Cincinnati, OH USA; 5 Department of Otolaryngology, University of Cincinnati Medical Center, Cincinnati, OH USA

**Keywords:** Endonasal, Endoscopic, Flap resuspension, Endonasal suturing, Barbed loop suture, Durastat system.

## Abstract

Suturing in the narrow endonasal corridor can be technically challenging. For some endonasal procedures, including inverted U-shaped nasopharyngeal flap (IUNF) harvest for cranio-cervical junction exposure, the ability to support meticulous flap resuspension offers clinical utility. Two novel suturing techniques offer promise: (1) use of barbed suture and (2) Durastat dural repair device, obviating the need for knot tying and endonasal needle driver/supination, respectively. Perform subjective and objective evaluation of IUNF resuspension with: (1) *running* mucosal suturing using endonasal needle driver and barbed, loop suture and (2) *interrupted* mucosal suturing using the Durastat device in 3 cadaveric heads. A IUNF was created and subsequently resuspended, (1) using a 3-0 monocryl barbed Stratafix with loop and endonasal needle driver. After Stratafix suture removal, (2) resuspension using interrupted sutures with the Durastat, spring loaded device was performed. Repair time was measured objectively. Suture precision was subjectively evaluated. Adequate IUNF resuspension was provided by both methods (3/3). The barbed suture required more procedural time on average (18:59 minutes), than the Durastat system (18:35 minutes). The small Durastat needle often failed to puncture the mucosal flap using spring mechanism alone, indicating that technical modification to increase needle size and spring torque could improve performance. Adequate mucosal resuspension was achieved in all specimens. Stratafix offered greater consistency with needle puncture and similar precision but was more technically challenging compared to the Durastat system. With appropriate technological modifications, the authors believe the Durastat system has greater potential to optimize IUNF resuspension.

**Supplementary Information:**

The online version contains supplementary material available at 10.1007/s10143-025-04068-x.

## Introduction

Endonasal exposure of the anterior C1 ring and ventral cranio-cervical junction (CVJ) has undergone marked evolution throughout the past 30 years [1–4]. Early reports of transnasal odontoidectomy involved resection of ventral nasopharyngeal tissue for CVJ exposure—relying on secondary intention for subsequent mucosal healing [5]. In ensuing years, adoption of a midline incision in the posterior nasopharyngeal tissue became increasingly common, although limitations with visualization were noted by some authors [6]. More recently, an inverted U-shaped nasopharyngeal incision has been introduced to provide more optimal visualization of the ventral CVJ [7]. However, in comparison to the limited midline nasopharyngeal incision, the inverted U-shaped nasopharyngeal incision is at greater risk of mucosal retraction and incisional diastasis resulting in delayed sinonasal healing. In this context, enhanced facility with endonasal mucosal resuspension offers hypothetical benefit. As procedures which necessitate proper mucosal reapproximation through the long and narrow endonasal corridor remain technically challenging. The aim of our study was to perform a pilot cadaveric analysis of the ability of two promising, innovative techniques to enhance endonasal mucosal resuspension.

## Materials and methods

Cadaveric dissections were performed in 3 embalmed cadaveric specimens. All dissections were performed under approved ethical guidelines for cadaveric research, and the study adhered to the principles of the Declaration of Helsinki. A 4-mm, 18-cm rigid Storz zero-degree endoscope was used for visualization.

All procedures were conducted by the senior author (JF). Briefly, the middle and inferior turbinates were lateralized and a posterior septectomy was performed. A red rubber catheter was used to retract the soft palate for enhanced visualization inferiorly. Lateral incisions were then made in the region of the Rosenmüller fossae, extending inferiorly to the palatal structures. A superior incision, just below the pharyngeal tubercle, connected the lateral incisions. The IUNF was elevated as a single layer, encompassing pharyngeal mucosa, constrictor muscles, and the longus capitis/colli musculature, using a curette for dissection and inferior retraction. Subperiosteal dissection facilitated visualization of the O-C1 joints. Measurements from Forbes et al. were referenced to avoid injury to critical adjacent neurovascular structures including hypoglossal nerves and internal carotid arteries [7]. Following appropriate exposure, the attention was turned to closure and mucosal resuspension. IUNF reconstruction was completed in two timed trials using: (1) *running* 3 − 0 Monocryl “knotless” Stratafix with loop suture in a standard fashion (followed by removal of suture material), and (2) *interrupted* closure using the Durastat spring-loaded repair dural device. Care was taken to prevent excessive tension on the sutures. In utilization of both techniques, the objective was a suture line free of gapping or other technical inadequacy. The timing of both techniques was documented for comparison, from first needle insertion into the flap to competition of knot tightening and suture cutting, and the quality of the resuspension was assessed in a binary fashion as “adequate” or “inadequate” by the senior author. Criteria for “inadequate resuspension” included evidence of gapping, ridging, or incomplete mucosal realignment.

## Results

**Fig. 1:**
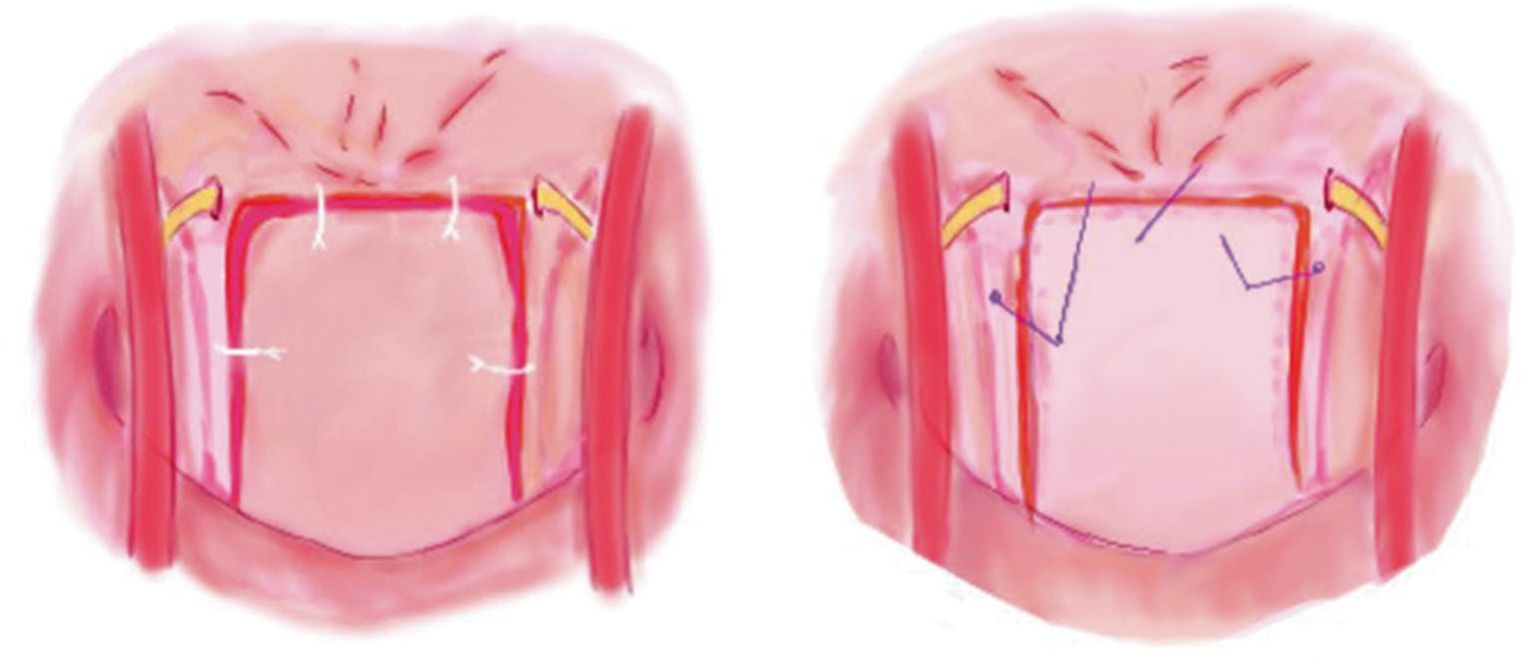
Endonasal (pictorial) images illustrating two techniques for resuspending the U-shaped nasopharyngeal flap. (**A**) Utilizing the Durastat system and a knot pusher to secure the knot. (**B**) Employing a barbed suture to eliminate the need for knot tying within the endonasal corridor

Mucosal reconstruction was successfully achieved in all specimens. The results are summarized in Tables 1 and 2. The average time required to resuspend the IUNF using the 3 − 0 Monocryl barbed Stratafix with loop and an endonasal needle driver was 18.59 min (SD ± 8.38, median 15.2), compared to the average time of 18.35 min (SD ± 1.82, median 19.27) using the Durastat system (Fig. 1). Suturing of the IUNF was felt to be more technically challenging in the Stratafix cohort; of interest, in this cohort, the procedural time decreased substantially with additional surgical experience (from trial 1 to 3). This trend suggests that initial complexity and unfami1iarity contributed to longer times early on, whereas efficiency improved with experience.

**Table 1:** Time comparing the use of 3 − 0 monocryl barbed stratafix with a loop and the durastat spring-loaded dural repair device

Specimen	Barbed loop suture	Durastat system
1	28.13	19.27
2	15.21	18.16
3	12.42	19.53
Min	12.42	18.16
Max	28.13	19.53
Median	15.21	19.27
Mean	18.59	18.35
SD	8.38	1.35

**Table 2:** Quality of IUNF resuspension using 3 − 0 monocryl barbed stratafix with a loop and the durastat spring-loaded dural repair device

Specimen	Barbed loop suture	Durastat system
1	A	A
2	A	A
3	A	A

**Fig. 2:**
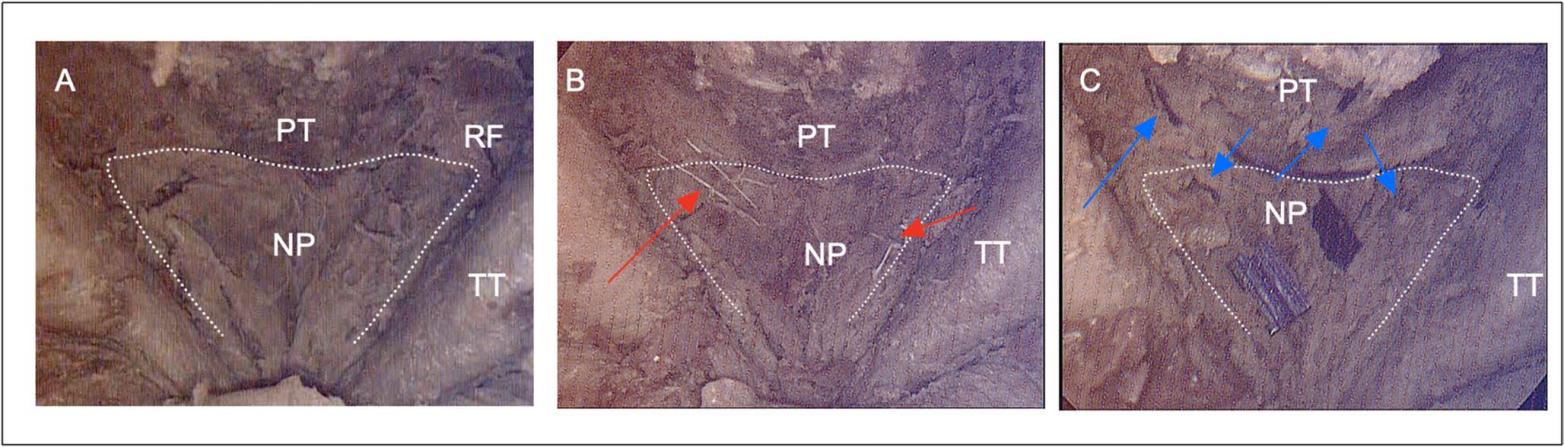
Intraoperative endoscopic endonasal images demonstrating relevant surgical anatomy and resuspension techniques in embalmed cadaveric specimens. Within the posterior nasopharynx (NP), several key anatomic landmarks can be easily visualized: pharyngeal tubercle (PT), Rosenmüller fossae (RF) and torus tubarius (TT). **A**. View of the inverted U-shaped nasopharyngeal flap. **B**. View of the IUNF resuspension using the Durastat spring-loaded repair dural device (red arrows). **C**. View of the IUNF resuspension using 3 − 0 monocryl barbed Stratafix with loop suture (blue arrows)

Conversely, in roughly one-third (30%) of the Durastat suture throws, the needle did not completely penetrate the mucosal tissue. This incomplete passage is a noteworthy technical limitation, as secure mucosal engagement is critical for hermetic closure and overall integrity of reconstruction. Failure of needle penetration likely related to inadequate needle design characteristics, such as small caliber and limited torque—features currently optimized for dural repair rather than for the thicker sinonasal mucosa. As a result, additional maneuvers were often required by the surgeon to fully advance using a separate endonasal needle driver. These additional maneuvers, which likely can be eliminated with improved design, artificially prolonged the surgical time associated with closure (Fig. 2).

## Illustrative case

**Fig. 3:**
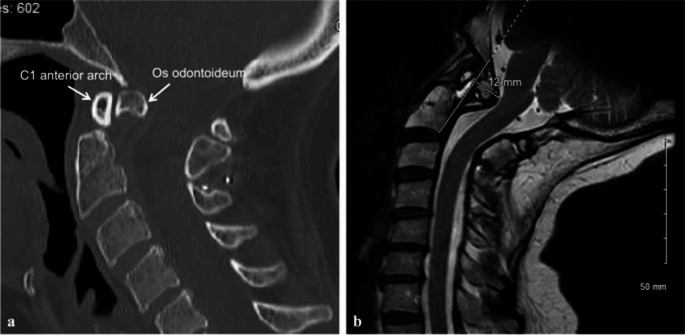
(**A**) Non-contrast cervical CT imaging demonstrated os odontoideum with retrolisthesis of C1 on C2 and dislodged wiring from previous surgery. (**B**) MRI demonstrated a Grabb-Oakes line measuring 12 mm with a ventral mass effect on the brainstem and spinal cord

A 64-year-old woman with os odontoideum presented with progressive cervicalgia, imbalance and worsening obstructive sleep apnea symptoms. Neurological examination revealed impaired tandem gait. Imaging demonstrated os odontoideum with C1 on C2 retrolisthesis, pseudarthrosis at C1–C2, dislodged wiring from prior surgery C1-2 stabilization, and a 12 mm Grabb-Oakes measurement with ventral compression of the brainstem and upper cervical spinal cord (Fig. 3). A two-stage endonasal odontoidectomy and revision of the C1-2 fusion was recommended.

The patient underwent trans-nasal endoscopic resection of the os odontoideum following harvest of the IUNF as described by Forbes et al [8]. The IUNF was created using a superior incision below the pharyngeal tubercle of the clivus and bilateral lateral incisions in the region of Rosenmuller fossae. The mucosal flap was transposed inferiorly using a 2 − 0 silk suture to provide surgical access while preserving vascularized tissue for reconstruction. Following resection of the anterosuperior arch of C1 and the os odontoideum, the IUNF was then mobilized from the oropharynx back to its normal position. Resuspension was performed using 4–0 Stratafix Monocryl sutures to secure the flap, followed by application of oxidized regenerated cellulose polymer Surgicel (Ethicon, Raritan, NJ) and Adherus hemostatic material. On postoperative day three, she underwent posterior wire removal and C1–C2 instrumented fusion using bilateral C1 lateral mass screws, a C2 pedicle screw placed on the right side and a translaminar screw on the left. The patient had an uneventful postoperative course and was discharged home on postoperative day two.

### Post-surgical outcome

**Fig. 4:**
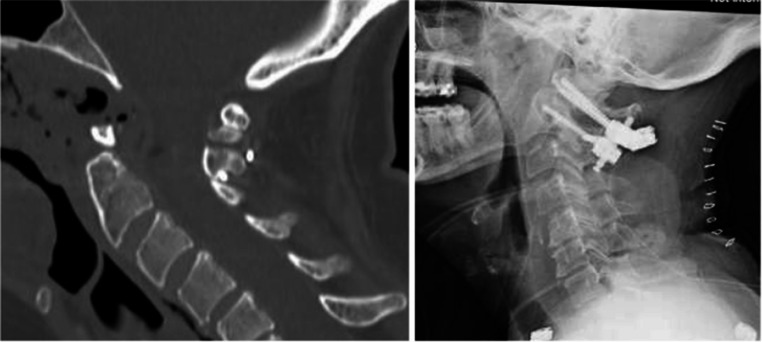
CT imaging anf plain film at three weeks post-op demonstrating staisfactory decompression and placement of hardware

**Fig. 5:**
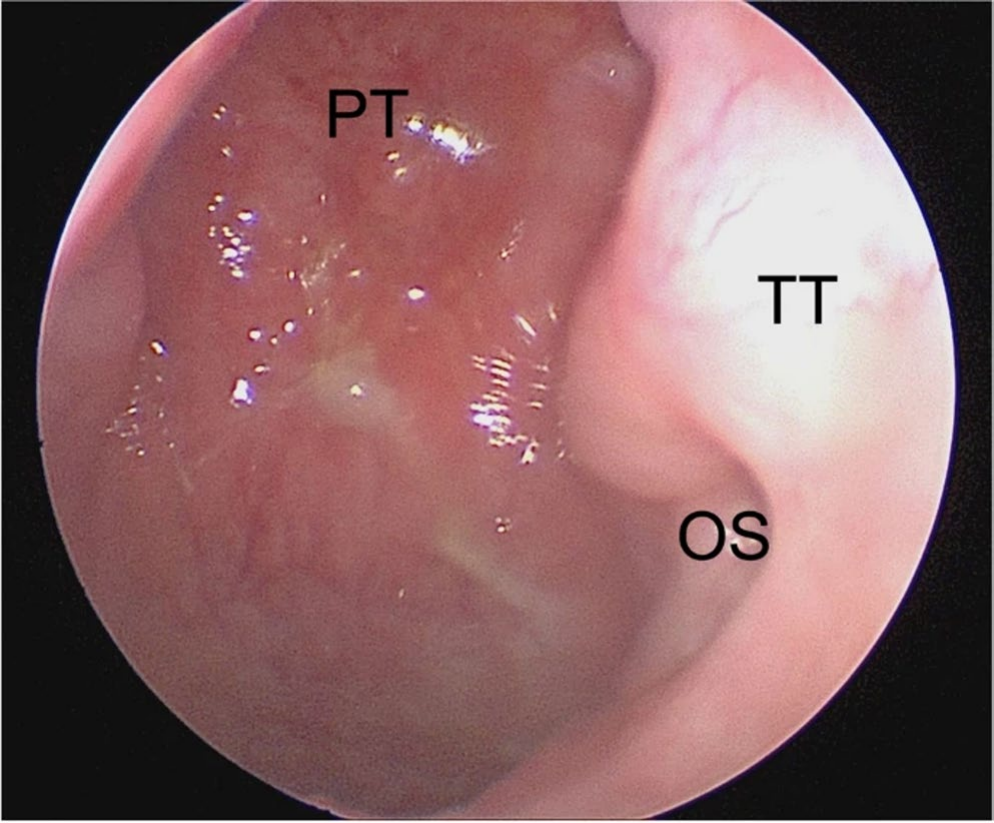
PT, pharyngeal tubercle; TT, torus tubarius; OS, os of the Eustachian tube; rigid nasal endoscopy at two-month post-op visit demonstrating a complete IUNF healing

At two-week follow-up, nasal endoscopy demonstrated appropriate healing of the nasopharyngeal mucosa. By three weeks post-operatively, the patient reported significant improvement in cervical pain with imaging confirming adequate decompression and satisfactory instrumentation positioning (Fig. 4). At the two-month otolaryngology follow-up, the IUNF was noted to have healed fully on endoscopic examination (Fig. 5). In the subacute post-operative period, the patient required bilateral myringotomy with tympanostomy tube placement due to middle ear effusions, possibly related to transient Eustachian tube dysfunction secondary to postoperative nasopharyngeal packing. At six months postoperatively, she reported substantial improvement in both her obstructive sleep apnea symptoms and cervicalgia. By nine months, the tympanostomy tubes had extruded spontaneously without recurrence of middle ear effusions. Her clinical course remained uneventful thereafter.

## Discussion

The primary objective of this study was to evaluate the feasibility and efficacy of resuspension of an inverted nasopharyngeal U-shaped flap using two innovative techniques: (1) barbed Stratafix suture and (2) the Durastat system. Despite several publications discussing technical nuances and outcomes following endonasal odontoidectomy, there is a paucity of information on the techniques and clinical utility of mucosal resuspension. In this pilot analysis, no significant difference in closure time and quality metrics between the barbed Stratafix and smooth Durastat system could be appreciated. Stratafix demonstrated dependable tissue anchoring and secure approximation, confirming its suitability for endonasal closure. The Durastat device also facilitated suitable closure. However, suboptimal physical characteristics of the Durastat device (presently configured for dural closure) likely artificially increased the time associated with closure. In this context, the authors felt the Durastat technique had greater future potential for IUNF resuspension.

Endonasal exposure of the anterior C1 ring and the cranio-cervical junction has undergone several procedural modifications since initial description. Recent publications have emphasized the U-shaped nasopharyngeal incision—which aims to optimize visualization of the ventral CVJ, and the potential to preserve tissue vascularity and promote faster nasopharyngeal healing with appropriate resuspension [7]. In this context, a need for innovative surgical methods able to support efficient and precise flap resuspension after harvest has been identified. The Stratafix barbed suture, originally developed in Somerville, New Jersey, USA for breast reconstruction and body contouring, offers key advantages by potentially reducing surgical site infections and eliminating the need for knot tying [9]. Conversely, the Durastat system, developed in Austin, Texas, USA for dural repair in the context of minimally invasive tubular exposure, does not require supination of the needle driver, which facilitates a more ergonomic repair.

In the long and narrow endonasal corridor, knot tying often requires utilization of a knot pusher. The longer distance and additional tools needed to fasten the knot translate to longer procedural times associated with closure. In contrast, the barbed Stratafix can be quickly passed through a loop after the initial throw and transected without a knot after the final throw—facilitating more efficient mucosal repair. However, barbed sutures, while effective for self-anchoring, carry a recognized risk of tissue trauma when excessive tension is applied. Their design concentrates force at the barb-tissue interface, which can exceed tissue resistance and lead to tearing, extrusion, or discomfort, particularly in fragile mucosa [10, 11]. As engineers continue to adapt barbed suture to increasingly smaller suture caliber, this technique may have utility that transcends to other neurosurgical procedures in the future.

In addition to considerations referable to endonasal knot tying, the movements of pronation and supination are comparably more challenging in the endonasal corridor [12]. The automated suture deployment mechanism of Durastat allows for consistent needle passage with the press of a button, eliminating the need for hand pronation or supination in tight spaces. In the context of repair of the dura in minimally invasive spinal procedures, the Durastat device has shown to reduce the time of repair by approximately 36%.^13^ By decreasing operating room time, minimizing the need for adjuncts, and decreasing the incidence of post-operative CSF fistula, previous studies have suggested Durastat offers cost savings for hospitals while improving patient outcomes for incidental durotomies [13]. As a final note, the smooth nonabsorbable monofilament polytetrafluoroethylene (PTFE) Durastat suture is likely to pose less of a risk to mucosal tearing than barbed suture analogues.

The presented illustrative case demonstrates the feasibility of mucosal resuspension using instruments that are currently available and approved for endonasal approaches. In this case, Eustachian tube dysfunction was investigated and attributed to excessive postoperative packing. While not encountered in the presented case, previous reports have described regional additional complications possibly attributable to suboptimal mucosal closure following odontoidectomy, including velopharyngeal insufficiency and hypernasal speech, as well as delayed sinonasal healing [14]. 

In this study, the small size and caliber of the Durastat needle, designed for dural closure, presented challenges for suturing of mucosal tissue within the endonasal corridor. The authors believe a larger and more robust needle would more reliably pierce the IUNF, possibly facilitating a more efficient closure. Additional research may be necessary to confirm that the Durastat spring loading mechanism consistently generates sufficient torque to pierce the mucosal layer of the IUNF with a redesigned needle. In the future, improved techniques for resuspension of the IUNF may have relevance for emerging investigational technology and decreasing morbidity associated with current technologies.

### Limitations

Limitations of this study include a relatively small sample size of cadaveric heads assessed, with only three embalmed cadaveric specimens evaluated, which limits statistical power and generalizability. The limited applicability of cadaveric tissue, as compared to living tissue, may not accurately replicate the biomechanical proprieties of living tissue, and possible degradation between sequential sutures could have influenced performance. Additional limitations include the fact that evaluation of mucosal repair was binary in nature (“adequate” or “inadequate”) and only performed by the senior author. These limitations were considered acceptable given the specified nature of this pilot analysis and the need for iterative refinement of the Durastat prototype prior to a more definitive analysis of mucosal resuspension. Futures studies with larger sample size, rigorous randomization, objective quantitative metrics will be essential to validate these early findings and establish clinical relevance.

## Conclusion

This study highlights the potential benefits of two innovative techniques, running suture with Stratafix and interrupted suture with Durastat, for endoscopic endonasal IUNF resuspension. Adequate mucosal reconstruction was successfully achieved in all specimens with both techniques. Stratafix offered more reliability with equivalent precision but was felt to be technically more challenging compared to the Durastat system. Of interest, the procedural time associated with Stratafix decreased substantially with progressive surgical experience (from trial 1 to 3). Resuspension with the Durastat system was technically quite simple. With appropriate technological modifications, the authors believe the Durastat system will likely become the superior option for mucosal resuspension in the future. The findings from this pilot study underscore the need for further research using larger cohorts, standardized outcome measures, and robust methodological frameworks to validate and build upon these initial observations.

## Supplementary Information

Below is the link to the electronic supplementary material.

Supplementary File 1 (mp4 331 MB)

Supplementary File 2 (mp4 261 MB)

## Data Availability

No datasets were generated or analysed during the current study.

## Declarations

### Clinical trial number

Clinical trial number not applicable.
